# Individuals with increased inflammatory response to ozone demonstrate muted signaling of immune cell trafficking pathways

**DOI:** 10.1186/1465-9921-13-89

**Published:** 2012-10-03

**Authors:** Rebecca C Fry, Julia E Rager, Haibo Zhou, Baiming Zou, June W Brickey, Jenny Ting, John C Lay, David B Peden, Neil E Alexis

**Affiliations:** 1 Department of Environmental Sciences and Engineering, Gillings School of Global Public Health, University of North Carolina, Chapel Hill, NC, USA; 2 Center for Environmental Medicine, Asthma, and Lung Biology, School of Medicine, University of North Carolina, Chapel Hill, NC, USA; 3 Biostatistics, Gillings School of Global Public Health, University of North Carolina, Chapel Hill, NC, USA; 4 Department of Microbiology and Immunology, School of Medicine, University of North Carolina at Chapel Hill, Chapel Hill, NC, USA; 5 Department of Pediatrics, University of North Carolina, School of Medicine University of North Carolina at Chapel Hill, Chapel Hill, NC, USA

**Keywords:** Air pollution, Environment, Ozone, Gene expression, Human sputum, Immune response, Innate immunity, Systems biology

## Abstract

**Background:**

Exposure to ozone activates innate immune function and causes neutrophilic (PMN) airway inflammation that in some individuals is robustly elevated. The interplay between immuno-inflammatory function and genomic signaling in those with heightened inflammatory responsiveness to ozone is not well understood.

**Objectives:**

Determine baseline predictors and post exposure discriminators for the immuno-inflammatory response to ozone in inflammatory responsive adult volunteers.

**Methods:**

Sputum induction was performed on 27 individuals before and after a two hour chamber exposure to 0.4 ppm ozone. Subjects were classified as inflammatory responders or non-responders to ozone based on their PMN response. Innate immune function, inflammatory cell and cytokine modulation and transcriptional signaling pathways were measured in sputum.

**Results:**

Post exposure, responders showed activated innate immune function (CD16: 31,004 MFI vs 8988 MFI; CD11b: 44,986 MFI vs 24,770 MFI; CD80: 2236 MFI vs 1506 MFI; IL-8: 37,603 pg/ml vs 2828 pg/ml; and IL-1β: 1380 pg/ml vs 318 pg/ml) with muted signaling of immune cell trafficking pathways. In contrast, non-responders displayed decreased innate immune activity (CD16, CD80; phagocytosis: 2 particles/PMN vs 4 particles/PMN) post exposure that was accompanied by a heightened signaling of immune cell trafficking pathways.

**Conclusions:**

Inflammatory responsive and non responsive individuals to ozone show an inverse relationship between immune cell trafficking and immuno-inflammatory functional responses to ozone. These distinct genomic signatures may further our understanding about ozone-induced morbidity in individuals with different levels of inflammatory responsiveness.

## Background

Ozone (O3) is an important criteria air pollutant known to induce exacerbation of asthma and other airway diseases [1,2]. Human challenge studies have shown that O3 exposure causes an immediate nociceptive decrease in lung function [3], increased airway hyperresponsiveness [3] and enhanced response to allergens in allergic volunteers [4,5]. O3 also induces airway inflammation in healthy and asthmatic volunteers that is marked by inflammatory cell (i.e. neutrophil (PMN)) influx to the airways [6,7]. In healthy volunteers, the inflammatory response in the airways can occur at O3 levels near (0.08 ppm) or below (0.06 ppm) the National Ambient Air Quality Standard (NAAQS) for ambient O3 with noted variability across individuals, such that some are very responsive while others have a very weak inflammatory response, suggesting the presence of a inflammatory response phenotype in humans [8,9]. Previous studies in healthy adults [10] as well as in animals [11] have suggested that certain genes, such as the GSTM1null genotype, can act as genetic modifiers of the inflammatory response to O3 and may explain some of the individual variation often observed. At present, the role inflammatory responsiveness plays in O3 susceptibility is unclear, and its association with ozone-induced changes in innate immune function and host defense genomic signatures remain largely unknown.

In this study, our goal was to characterize factors in the airways that may contribute to the differential response of adult volunteers to ozone. Various cellular features associated with ozone responsiveness in the airways were assessed including the inflammatory response, innate immune function and transcriptional signaling both before and after ozone exposure. We stratified a cohort of 13 healthy, 4 atopic and 10 allergic asthmatic subjects on the basis of their PMN response to O3 into a responder and non-responder group. The inflammatory response was defined as the difference in % PMNs pre versus post exposure, and a PMN response of >10% successfully distinguished responders from non-responders as previously reported [12-14]. Our analysis focused on establishing cell-associated differences in sputum at baseline and post exposure in inflammatory responders and non-responders. Cells recovered from sputum derive from the lumen of the large central airways and thus reflect pathophysiologic cellular events in the large airways [15]. Specifically, we analyzed (1) genomic signatures, (2) biological indicators of innate immune function and (3) cellular and fluid phase markers of inflammation in order to determine whether these indicators were constitutively predictive at baseline and/or discriminatory post exposure of the inflammatory responder phenotype in adult volunteers. Furthermore, as it is unclear whether asthma or atopy status is associated with the inflammatory response phenotype, we examined the relationship of asthma and atopy status in our cohort of responders and non-responders.

We demonstrate that ozone responders and non-responders can be differentiated both at baseline and post exposure using various cellular indicators. Most strikingly, we find that ozone-induced activation of innate immune function and inflammation is negatively associated with genomic signaling of immune cell trafficking networks. Specifically, responders and non responders have opposite genomic responses in terms of activation of immune cell trafficking. In addition, we show that responders compared to non responders have elevated expression of pro-inflammatory cytokines IL-8 and Il-1b at baseline, suggesting a primed inflammatory state, but simultaneously possess muted innate immune function. Asthma or atopy status per se does not influence the inflammatory response phenotype.

## Methods

### Subjects

Twenty seven adult subjects (13 healthy, 4 allergic non asthmatics, 10 allergic asthmatics), aged 21 – 35 years, with no history of smoking in the past 10 years, completed the study (Table 1). All subjects underwent a physical examination, a routine blood panel with complete blood cell count, and differential and allergy skin testing for common allergens (cat, cockroach, dust, grass, mite, shellfish, tree, weeds). Healthy subjects were required to have a negative methacholine challenge test result, and asthma status was confirmed by clinical history and positive (≥8 mg/ml) methacholine challenge test. No asthmatics were receiving oral or inhaled corticosteroid therapy but all used albuterol on an as needed basis. Female subjects were required to have a negative urine pregnancy test result before challenge, and all volunteers were required to be free of chronic cardiovascular or respiratory illness (excluding asthmatics) and be free of acute respiratory illness (for asthmatics, no exacerbations requiring oral or inhaled corticosteroids) within 4 weeks of O3 challenge. All subjects had FEV_1_ and forced vital capacity (FVC) values of 80% or greater of predicted value and FEV_1_/FVC ratios of 75% or greater of predicted normal value for height and age. All subjects were screened for their ability to provide an adequate induced sputum sample during their training sessions 48 hours prior to exposure and this sample acted as their pre-exposure baseline. Pre and post sputum samples were separated by a minimum of 48 hours. The study protocol was approved by the Institutional Review Board at the University of North Carolina Medical School in Chapel Hill and the US Environmental Protection Agency (EPA), and informed consent was obtained from all subjects before their participation in the study.

**Table 1:** Subject demographic data

**Responders**	**Height (cm)**	**Weight (kg)**	**Age (yr)**	**Gender**	**Race**	**Smoker**	**Diagnosis**	**Minute ventilation (Vmin)**
**01**	157	61.2	21	Female	Caucasion	no	healthy	36.8
**02**	195	81.8	20	Male	Caucasion	no	healthy	29.5
**03**	171	53.8	30	Male	Caucasion	no	healthy	28.3
**04**	180	83	31	Male	Asian	no	healthy	27.3
**05**	182	78.6	23	Male	Caucasion	no	healthy	37.3
**06**	170	58.5	28	Female	Caucasion	no	healthy	35.5
**07**	153	48.7	22	Female	Caucasion	no	healthy	30
**08**	159	75.4	22	Female	Caucasion	no	healthy	32
**09**	186	66.6	23	Male	Caucasion	no	atopic + ve skin test	32.8
**10**	167	77.4	35	Male	African American	no	atopic + ve skin test	34
**11**	173.5	82.5	22	Male	Caucasion	no	atopic + ve skin test	31.3
**12**	168	92.3	39	Female	Caucasion	no	atopic asthmatic	34.3
**13**	181	75.1	20	Male	allergic asthmatic	no	atopic asthmatic	33.3
**14**	168	97.3	20	Female	allergic asthmatic	no	atopic asthmatic	30.4
**15**	178	74.2	31	Female	Caucasion	no	atopic asthmatic	35
**16**	167	69.3	27	Female	Caucasion	no	atopic asthmatic	32.5
**17**	170	52	22	Female	Caucasion	no	atopic asthmatic	30.8
**18**	165	73.4	25	Female	Caucasion	no	atopic asthmatic	31.5
**Mean (+/− SEM)**	**172 (3)**	**72 (3)**	**26 (1)**					**33 (1)**
**Non-Responders**								
**01**	193	94.8	26	Male	Caucasion	no	healthy	32.1
**02**	156	56.3	27	Female	Caucasion	no	healthy	30.8
**03**	162	59.1	25	Female	Caucasion	no	healthy	34.8
**04**	172.25	70.9	24	Female	Caucasion	no	healthy	33.8
**05**	157	77.9	26	Female	Caucasion	no	healthy	28.3
**06**	158	68.4	20	Male	Caucasion	no	atopic + ve skin test	32.3
**07**	155	60.6	25	Female	Caucasion	no	atopic asthmatic	33
**08**	173	64.8	27	Male	Causasion	no	atopic asthmatic	31.8
**09**	178	64	20	Male	Caucasion	no	atopic asthmatic	33.5
**Mean (+/− SEM)**	**167 (4)**	**69 (4)**	**24 (1)**					**32 (1)**

### Ozone (O3) exposure and testing protocol

The O3 exposures were conducted in an exposure chamber at the EPA Human Studies Facility on the campus of the University of North Carolina (Chapel Hill, NC). Each subject was exposed to 0.4 ppm O3 for two hours while performing four 15-minute sessions of intermittent moderate exercise (expiratory minute ventilation, 30–40 L/min) on a treadmill, separated by 15 minutes of seated rest. Lung function, breath sounds, and vital signs were assessed immediately before and after exposure.

### Sputum collection and analysis

Sputum was obtained 5 hours after exposure and processed as previously described [10]. Sputum samples were used as O3 preferentially absorbs in the large central airways during exercise [16], the same region from which sputum samples originate [15]. In brief, three 7-minute inhalation periods of nebulized hypertonic saline (3%, 4%, and 5%; UltraNeb 99 ultrasonic nebulizer; DeVilbiss, Jackson, TN) were followed by expectoration of sputum into a sterile specimen cup. Cell-rich “plug” material was selected from the raw sample and treated with a dilute (0.1%) solution of dithiothreitol (Sputolysin; Calbiochem, San Diego, CA) in Dulbecco PBS. Aliquots of sputum supernatant were collected and immediately frozen and stored at −80°C for future cytokine measurement, and the cell pellet was analyzed for cell viability (trypan blue exclusion), total cell count and differential leukocyte counts (Hema-Stain-3; Fisher Scientific, Hampton, NH). A portion of the remaining cells were also used for cell surface immunophenotyping (CD11b, mCD14, HLA-DR, CD16, CD80, CD86) as assessed by flow cytometry as previously described [10]. Acquired sputum samples considered acceptable for processing had a minimum of 75 mg of selected plug material, cell viability greater than 50%, and squamous epithelial cells less than 40%. All sample processing, including flow cytometric assays and slide preparations, were performed on the same day of collection. Flow analysis was performed on fixed samples (0.5% paraformaldehyde) within 24 hours of acquisition on a BD LSR-II flow cytometer (BD Immunocytometry Systems, San Jose, CA).

Cytokines were measured using multi-plex technology (Meso ScaleDiscovery/MSD, Gaitherburg, MD). Each sample was analyzed with the Human MIP-1 alpha Ultra Sensitive Kit (lot no. K0031370) and the Human TH1/TH2 10-Plex Ultra Sensitive Kit (lot no. K0031431). All supernatant samples were diluted 1:4 and had a final dithiothreitol (DTT) concentration of less than 1 mmol/L where no deleterious effects from the DTT have been observed with the MSD platform.

Functional assays for oxidative burst activity and phagocytosis of opsonized zymosan were also assessed on sputum inflammatory cells by means of flow cytometry as previously described [10]. Several phagocytosis parameters were assessed including the percentage of internalized (vs externally adhered) zymosan particles using Trypan Blue stain as the quenching reagent.

### Classification of the ozone induced inflammatory response

**Figure 1:**
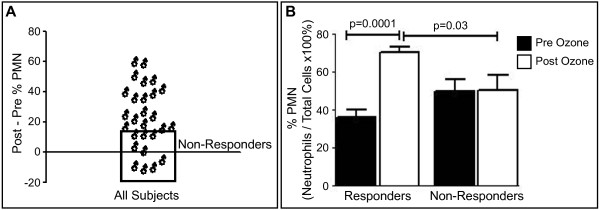
**Ozone response for neutrophils differs between responders and non-responders.** (**A**) Inflammatory non-responders and responders are distinguished on the basis of their PMN response (% PMN post -% PMN pre exposure) using an ozone response cutoff level of less than 12 for non-responders and greater than 12 for responders. (**B**) The pre versus post exposure % PMN levels differ significantly (p < 0.05) for responders with ozone exposure, as well as the responders versus non-responders post ozone exposure.

Subjects demonstrated a range of responsiveness with respect to airway neutrophil proportion (%) following ozone exposure (Figure 1A). Inflammatory responsiveness was defined as the % PMN post -% PMN pre O3 exposure. In general, individuals could be grouped as those with a minimal inflammatory response or “non-responders” (N = 9) and those with a robust inflammatory response or “responders” (N = 18). An inflammatory response (% PMN post -% PMN pre O3) ≥ 12 was used to distinguish inflammatory responders and non-responders and is a similar criteria as used previously by our group and others [12-14]. The mean ozone “response” for all endpoints is defined as the mean of the sum of the individual subject differences between post and pre exposure.

### FEV1 response

Spirometry was performed on a 10.2-L dry seal digital spirometer interfaced to a computer (SensorMedics Model 1022; SensorMedics; Palm Springs, CA). At least three sets of qualified data were obtained and the largest value selected for FEV_1_ and FVC as per American Thoracic Society guidelines [17]. Pulmonary function on all subjects was measured using one dedicated spirometer and by one certified pulmonary function technician to minimize variability. Measurements were performed before and immediately after exposure for use in endpoint analysis.

### RNA Labeling

Total sputum RNA was prepared with Trizol (Invitrogen, Carlsbad, CA) homogenization and QiagenRNeasy column purification (Valencia, CA) according to manufacturer’s protocols. RNA integrity was assessed by comparing 18S and 28S rRNA species using a Bioanalyzer (Agilent Technologies, Inc., Santa Clara, CA). RNAs from each group were labeled by amino-allyl nucleotide incorporation and reverse transcription with SuperScriptIII (Invitrogen) according to manufacturer’s protocol (Epicentre Biotechnologies, Madison, WI). Probes were then coupled to fluorescent-labeled cy dyes (GE Healthcare, Piscataway, NJ), such that the experimental samples were Cy5 (red) labeled while the human universal reference RNA (Stratagene/Agilent) sample was Cy3 (green) labeled.

### Microarray analysis

Each Cy5 sample aRNA probe was paired with Cy3 reference aRNA probe and hybridized to microarrays in formamide-containing buffer overnight at 42°C using a Maui Hybridization System (BioMicro Systems Inc., Salt Lake City, UT). The custom-designed microarray consisted of 70-mer oligonucleotides representing more than 2000 immune response gene targets. Commercially available and custom-designed oligos were purchased from Operon Biotechnologies, Inc. (Huntsville, AL) and printed onto glass slides by the Duke Microarray Facility (Duke University, Durham, NC). Each microarray contained targets replicate-spotted four times. The gene target pool also included housekeeping genes (i.e. *ACTB*, *GAPD*, *TUBA*, ribosomal proteins *RPL13A*, *RPS9*) used as internal references, negative background controls and known “spiked-in” RNA oligos (Applied Biosystems, Austin, TX). Technical replicate assays were performed.

### Microarray data analysis

The hybridized spot intensities were acquired using a GenePix 4000B scanner (Molecular Devices Corp, Sunnyvale, CA) and assessed using GenePix Pro 6.0 software. Median pixel intensities (with background subtracted) were uploaded in GeneSpring 10 (Agilent Technologies, Inc., Santa Clara, CA), normalized by global LOWESS fit [18], log base 2 transformed, filtered so as to exceed the background and spot quality criteria (i.e.“Absent” probes were not included) and merged into datasets. Gene lists and hierarchical clusters of differentially expressed genes were generated. The use of R software through Bioconductor was examined to analyze hybridization signal intensities [19,20]. To control for multiple comparison, we used false discovery rate (FDR), estimated by 5000 permutations for each gene.

### Network analysis and enriched canonical pathways

Molecular network and enriched canonical pathway analysis was performed using the Ingenuity database (Ingenuity Systems, http://www.ingenuity.com, Redwood City, CA). The Ingenuity database provides a collection of gene to phenotype associations, molecular interactions, regulatory events, and chemical knowledge accumulated to develop a global molecular network. The lists of differentially expressed mRNAs were overlaid onto this global molecular network, where protein networks strongly associated with the targets were algorithmically constructed based on connectivity. Statistical significance of each network was calculated using a Fisher’s exact test. This test generated a p value representing the interconnectivity between genes, indicating the likelihood of the gene products interacting by chance alone. Enriched canonical pathways within these networks were also identified, where a Fischer’s exact test was used to identify significantly associated canonical pathways. Canonical pathways with p < 0.01 were assessed.

### Statistical methods

Baseline responses and O3 responses for all phenotype endpoints were compared using a Student’s T-test (using Prism version 5.01). In cases where data were not normally distributed, non-parametric tests (Mann Whitney) were used. The relationship between O3-induced inflammatory response and asthma status was analyzed using an exact chi-square test and the Mantel-Haenszel chi-square using S.A.S. (S.A.S, version 9.2). A p value of <0.05 was considered statistically significant.

## Results

### Inflammatory (PMN) response phenotype: ozone (O3) responders and non-responders

Sputum samples were collected from 27 volunteers exposed to 0.4 ppm O3 for two hours. Differential cell count analysis showed non-responders (N = 9) to have a minimal PMN response of 0.6% (±3%), while responders (N = 18) had a significant (p = 0.0001) PMN response of 35% (±3%) (Figure 1A). Post exposure, PMN levels were significantly (p = 0.03) higher in responders compared to non-responders as expected, while baseline % PMN levels did not differ between inflammatory responders and non-responders (Figure 1B). We note, of the 27 total subjects, 10 were asthmatic and 4 were atopic without asthma. The inflammatory response however was not associated with either atopy or asthma as measured by chi square analysis (p = 0.69). This was further confirmed when the subjects’ neutrophil response to ozone were stratified by disease group and showed no difference when analyzed by ANOVA (p = 0.9).

### FEV1 response in inflammatory responders and non-responders

Percent predicted FEV1 (%) and the “% predicted FEV1 response” were measured to determine whether lung function was different at baseline and post ozone exposure in inflammatory responders and non responders. The mean FEV1 “response” was calculated as the mean of the sum of the individual subject differences between post and pre % predicted FEV1. We report that the % predicted FEV1 was not significantly different between inflammatory responders and non responders, respectively at pre exposure (106 ± 4% vs 107 ± 4%, p > 0.05) and post exposure (91 ± 4% vs 93 ± 5%, p > 0.05). The % predicted FEV1 “response” was also not significantly different between responders and non responders (data not shown).

### Specific cytokines increased at baseline and post exposure

In order to characterize cytokine responses to O3 exposure in inflammatory responders and non-responders, the levels of eight different cytokines were measured pre and post exposure (Table 2). At pre exposure, IL-8 was significantly elevated in responders compared to non-responders. At post exposure, IL-1β and IL-8 were significantly increased in responders compared to non-responders. Responders also demonstrated a significantly greater increase from pre exposure in IL-8 and IL-1β than non-responders (data not shown). All other cytokines (IL-2, IL-6, TNFα, INFγ, IL-12) remained at relatively stable levels pre or post exposure for both responders and non-responders.

**Table 2:** Cytokine levels pre and post ozone exposure

**Cytokine (Pg/ml)**	**Responders pre exposure**	**Responders post exposure**	**Non responders pre exposure**	**Non responders post exposure**
**IL-1β**	989 (511)	1380 (672)**	510 (184)	318 (95)
**IL-8**	20234 (10347)*	37603 (12024)**	3161 (1567)	2828 (1076)
**IL-6**	104 (38)	304 (89)	343 (152)	348 (138)
**IL-10**	28 (14)	100 (62)	55 (37)	16 (9)
**TNFα**	223 (142)	141 (49)	44 (14)	63 (32)
**INFγ**	204 (56)	272 (74)	105 (37)	138 (51)
**IL-12**	186 (67)	210 (104)	209 (87)	133 (44)
**IL-2**	203 (50)	219 (69)	440 (164)	305 (101)

Mean cytokine levels are shown (+/− standard error).

Pg/ml = picograms per mililiter.

* p < 0.05 Responder vs Non Responder pre-exposure; ** p < 0.05 Responder vs Non Responder post-exposure Pre Exposure = 48 hours pre exposure; Post Exposure = 5 hours post exposure.

### Modified cell surface phenotype expression in inflammatory responders and non-responders

Expression, measured as mean fluorescence intensity (MFI), of cell surface phenotypes on sputum monocytes, macrophages, neutrophils and dendritic cells was assessed in inflammatory responders and non-responders pre and post O3 exposure. The mean O3 “response” for cell surface phenotypes was calculated as the mean of the sum of the individual subject differences between post and pre exposure MFI values (Table 3). Pre exposure, there were significant (p < 0.05) differences in surface marker expression between responders and non-responders for CD14 and CD80 on monocytes and CD86 on macrophages. Post exposure, significant differences in expression between responders and non responders were observed for CD16, CD80, HLA-DR and CD11b (trend) on macrophages, and HLA-DR on monocytes. When the mean O3 “response” was compared between responders and non-responders, significant (p < 0.05) differences were found for macrophage CD11b expression and monocyte CD80 expression. No other significant differences between responders and non-responders were found at pre or post exposure or for the calculated O3 “response” for the other surface phenotypes measured.

**Table 3:** Cell surface phenotype expression pre and post ozone exposure

**Surface marker (MFI)**	**R pre ozone**	**R post ozone**	R ozone response*	**NR pre ozone**	**NR post ozone**	NR ozone response*	**Pre exposure R vs NR**	**Post exposure R vs NR**	**Ozone response R vs NR**
**CD11b Mac**	23964 (4448)	44986 (9539)	110 (42)	37861 (11770)	24770 (4280)	8 (30)	ns	P = 0.07	p = 0.05
**CD14 Mono**	5776 (559)	11800 (1082)	149 (41)	9886 (1016)	19348 (4647)	92 (36)	p = 0.004	ns	ns
**HLA-DR Mono**	7399 (849)	10611 (832)	85 (35)	9715 (1412)	15361 (2110)	88 (45)	ns	P = 0.02	ns
**HLA-DR Mac**	46144 (3293)	48527 (5232)	9 (13)	60440 (15219)	76424 (9254)	56 (29)	ns	P = 0.02	ns
**CD16 Mac**	21259 (6465)	31004 (8624)	100 (38)	8030 (2266)	8988 (2011)	27 (30)	ns	P = 0.01	ns
**CD80 Mono**	2956 (603)	2236 (576)	16 (49)	825 (370)	1506 (414)	648 (572)	p = 0.01	ns	p = 0.01
**CD80 Mac**	3202 (611)	5178 (1116)	175 (88)	3440 (1642)	2014 (649)	32 (84)	ns	P = 0.02	ns
**CD86 Mac**	26012 (2577)	21996 (2422)	172 (193)	42833 (12057)	42135 (9157)	13 (22)	p = 0.05	ns	ns
**CD11b Mono**	31267 (4186)	32517 (3789)	34 (25)	27031 (3957)	35935 (6247)	47 (25)	ns	ns	ns
**CD11b PMN**	48975 (3856)	53935 (6220)	18 (16)	51808 (8185)	59310 (9073)	27 (25)	ns	ns	ns
**CD14 Mac**	7937 (975)	9516 (973)	66 (50)	18093 (10437)	12001 (2838)	9 (14)	ns	ns	ns
**HLA-DR DC**	27304 (2787)	27230 (2087)	6 (8)	25986 (3583)	29416 (2101)	28 (25)	ns	ns	ns
**CD16 Mono**	9422 (1763)	7048 (926)	11 (21)	9511 (4755)	6801 (1847)	15 (27)	ns	ns	ns
**CD16 PMN**	64160 (10852)	62617 (9331)	23 (25)	65491 (29381)	81816 (26611)	65 (30)	ns	ns	ns
**CD86 Mono**	6768 (893)	6835 (374)	36 (25)	6973 (895)	10588 (10588)	75 (61)	ns	ns	ns

Mean (+/− standard error) values are shown for each cell surface marker, along with the corresponding cell type.

R = Responders; NR = Non-Responders; MFI = mean fluorescence intensity; Mono = monocyte; Mac = macrophage; PMN = Polymorphonuclear neutrophil; ns = not significant.

* Ozone Response values were calculated as the mean of the sum of the individual subject differences in expression (MFI) between post and pre exposure.pre = 48 hours pre exposure; post = 5 hours post exposure.

### Oxidative burst activity is not changed by ozone

Oxidative burst activity was evaluated pre and post O3 exposure using a functional assay. O3-induced changes in oxidative burst activity were not significantly different between inflammatory responders and non-responders (Table 4). Furthermore, baseline (pre O3) oxidative burst activity did not significantly differ between responders and non-responders (Table 4).

**Table 4:** Oxidative burst activity pre and post ozone exposure

**Oxidative burst activity (MFI/%)**	**R pre ozone**	**R post ozone**	**R ozone response**	**NR pre ozone**	**NR post ozone**	NR ozone response*	**Pre exposure R vs NR**	**Post exposure R vs NR**	**Ozone response R vs NR**
**Mac**	118 (16)	84 (15)	−19 (15)	114 (25)	128 (31)	116 (112)	ns	ns	ns
**% Mac**	55 (8)	36 (7)	−20 (20)	52 (12)	36 (13)	−18 (29)	ns	ns	ns
**PMN**	41 (7)	35 (5)	14 (23)	49 (11)	63 (13)	127 (134)	ns	ns	ns
**% PMN**	73 (8)	58 (7)	35 (58)	65 (11)	49 (17)	−4 (37)	ns	ns	ns

Mean (+/− standard error) values are displayed for burst activity (MFI) and % of cells undergoing burst response.

R = Responders; NR = Non-Responders; MFI = mean fluorescence intensity; Mac = macrophages; PMN = Polymorphonuclear neutrophil; ns = not significant.

* Ozone Response values were calculated as the mean of the sum of the individual subject differences in oxyburst values between post and pre exposure.

pre = 48 hours pre exposure; post = 5 hours post exposure.

### Phagocytosis activity varies between inflammatory responders and non-responders

Phagocytosis, assessed in sputum macrophages and neutrophils, was expressed as total phagocytic capacity (MFI), the percentage of cells undergoing phagocytosis, the percentage of internalized particles and the internal to external ratio (I:E ratio) of particle uptake by cells (Table 5). These indicators were evaluated pre and post ozone exposure. As shown in Table 5, several phagocytosis indices were significantly (p < 0.05) different between inflammatory responders and non-responders at baseline in both macrophages and neutrophils. Specifically, responders had a decrease in all phagocytosis endpoints in macrophages and lower particle internalization in neutrophils compared to non-responders. Responders did however, show higher total phagocytic capacity (MFI) in neutrophils suggesting greater particle adherence to the neutrophil surface but importantly, a decreased capacity to engulf and internalize the particle. Post exposure, neutrophil phagocytosis was significantly increased in responders versus non responders for total phagocytic capacity and for the number of particles taken up per neutrophil (Table 5). The mean O3 “response” (mean of the sum of the individual subject differences in phagocytosis between post and pre exposure) by macrophages was not different between responders and non-responders but did differsignificantly for neutrophils across several indices. As shown in Table 5, responders’ neutrophils had significantly increased internalization capacity following O3 exposure compared to non-responders who showed decreased internalization and a decrease in the percentage of neutrophils undergoing phagocytosis. Altogether, phagocytosis activity greatly differed between inflammatory responders and non-responders at baseline for both macrophages and neutrophils, while neutrophils were specifically affected post O3.

**Table 5:** Phagocytosis activity pre and post ozone exposure

**Phagocytosis**	**R pre ozone**	**R post ozone**	R ozone response*	**NR pre ozone**	**NR post ozone**	NR ozone response*	**Pre exposure R vs NR**	**Post exposure R vs NR**	**Ozone response R vs NR**
**Mac (MFI)**	443 (140)	660 (138)	139 (59)	412 (167)	555 (234)	91 (50)	ns	ns	ns
**% Mac**	34 (4)	35 (4)	17 (15)	51 (6)	44 (8)	−14 (13)	p = 0.03	ns	ns
**Particles/Mac**	3 (1)	4 (1)	138 (59)	3 (1)	4 (2)	91 (50)	ns	ns	ns
**% Internalized Mac**	31 (5)	32 (4)	26 (19)	64 (10)	57 (17)	−19 (21)	p = 0.02	ns	ns
**Internal:External Particle Ratio Mac**	0.6 (0.2)	0.5 (0.1)	40 (27)	2.1 (0.6)	2.3 (0.9)	−7 (42)	p = 0.02	ns	ns
**PMN (MFI)**	565 (144)	542 (105)	61 (41)	197 (62)	226 (27)	78 (62)	p = 0.03	p = 0.01	ns
**% PMN**	26 (4)	40 (6)	70 (26)	43 (5)	33 (2)	−18 (14)	p = 0.01	ns	p = 0.01
**Particles/PMN**	4 (1)	4 (1)	66 (44)	1 (0.3)	2 (0.2)	93 (58)	p = 0.03	p = 0.02	ns
**% Internalized PMN**	29 (4)	46 (5)	95 (37)	72 (15)	65 (14)	−5 (13)	p = 0.03	ns	p = 0.03
**Internal: External Particle Ratio PMN**	0.5 (0.1)	1 (0.2)	196 (73)	6 (3)	3 (2)	−6 (45)	p = 0.03	ns	p = 0.04

Mean (+/− standard error) values are displayed for each phagocytic indicator.

R = Responders; NR = Non-Responders; MFI = mean fluorescence intensity; Mac = macrophage; PMN = polymorphonuclear neutrophil; ns = not significant.

* Ozone Response values were calculated as the mean of the sum of the individual subject differences in phagocytosis values between post and pre exposure.pre = 48 hours pre exposure; post = 5 hours post exposure.

### Large differences in genomic response between inflammatory responders and non-responders

**Figure 2:**
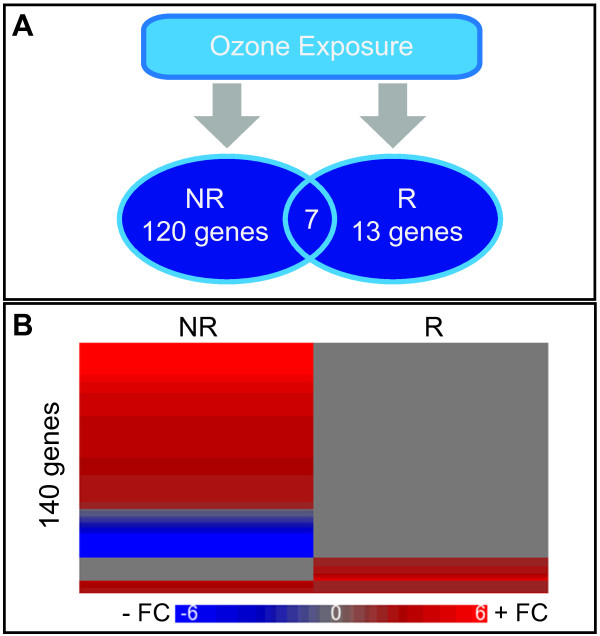
**Ozone exposure causes a varied genomic response between inflammatory responders and non-responders.** (**A**) Human ozone exposure induced significant changes in the expression of genes, largely within the NR group. (**B**) A heat map illustrates the 140 ozone-modulated genes identified as differentially expressed in at least one subject group exposed to ozone. Blue indicates negative fold change (FC) in expression (post ozone challenge / baseline), and red indicates positive FC.

A total of 140 genes were identified with significantly altered expression following O3 (see Additional file 1). Of these, 120 genes were modulated in the non-responder group, 13 genes were modulated in the responder group, and an additional 7 genes were changed in both groups (Figure 2A). The majority of the altered genes had increased expression levels after O3 exposure (Figure 2B).

To gain further insight into whether the alterations in gene expression profiles occurred simply as a result of changes in cell populations, the lists of ozone-modulated genes in each group were compared to a previously published list of 411 genes that have been suggested to be granulocyte-specific in comparison to other types of leukocytes [21]. Comparing the 13 genes modulated by ozone in the inflammatory responder group to the granulocyte-specific genes revealed one gene, neutrophil cytosolic factor 2 (*NCF2*), which may have been measured at increased expression levels resulting from the increased presence of neutrophils found in the responder group. However, this gene also showed increased expression in the non-responder group which did not show significant changes in neutrophil cell counts. This comparison gives evidence to suggest that the changes in genomic signatures induced by ozone do not reflect changes in neutrophil cell count.

**Figure 3:**
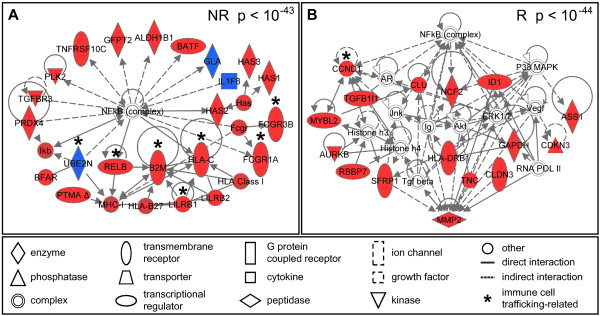
**Differences in genomic signaling networks between inflammatory responders and non-responders.** The most significant networks associated with ozone-induced gene expression changes in (**A**) inflammatory non-responders and (**B**) inflammatory responders are displayed. Networks are shown with symbols representing products of genes that are up-regulated (red symbols) by ozone, down-regulated (blue symbols) by ozone, or are associated (clear symbols) with the differentially expressed genes. Genes that encode proteins that play a role in immune cell trafficking are designated with an asterisk (*).

**Figure 4:**
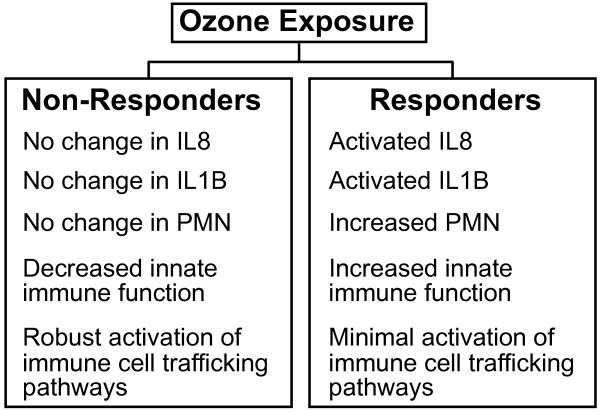
Summary of the ozone-induced changes in non-responders and responders.

Biological networks potentially affected by O3 exposure were constructed using the genes that showed altered expression levels in responders and non-responders. Nine networks were identified using the genes modified by O3 exposure in non-responders, while only two networks were identified in the responders (see Additional files 2 and 3). Figure 3A and 3B show the biological networks with the highest statistical significance for inflammatory non-responders and responders, respectively. While no genes are shared between the two most significant networks, both contain the common transcriptional regulator nuclear factor of kappa light chain gene enhancer in B cells (NF-κB). An examination of the functional role of the integrated proteins identifies a difference in the number of genes that encode immune cell trafficking-associated proteins. Specifically, the non-responders showed increased expression levels of 7 immune cell trafficking-associated genes while responders had a muted response with only 1 immune cell tracking-associated gene (Figure 3A,B). In particular, ubiquitin-conjugating enzyme E2N, (*UBE2N*), v-rel reticuloendotheliosis viral oncogene homolog B, (*RELB*), beta-2-microglobulin, (*B2M*), major histocompatibility complex, class I, C, (*HLA-C*), leukocyte immunoglobulin-like receptor, subfamily B (with TM and ITIM domains), member 1, (*LILRB1*), fragment of IgG, high affinity Ia, receptor (CD64), (*FCGR1AFc*), fragment of IgG, low affinity IIIb, receptor (CD16b), (*FCGR3BFc*), and cyclin D1, (*CCND1*) are genes that encode proteins involved in immune cell trafficking. It should also be noted that *FCGR3B* is a gene that is highly expressed in neutrophils [21]. Taken together, inflammatory responders displayed activated markers of inflammation that included IL-8, II-1b and increased levels of PMN, enhanced immune cell function and suppressed expression of immune cell trafficking transcripts. In contrast, non-responders demonstrated unchanged markers of inflammation, activated immune cell function and up-regulated immune cell trafficking transcripts (Figure 4).

## Discussion

We set out to identify whether cellular and biochemical indicators in sputum collected both at baseline and post exposure could predict and characterize the inter-individual differences in adult volunteers who were grouped according to their level of inflammatory responsiveness to ozone. Specifically, we examined whether markers of innate immune function and targeted transcripts for immune cell trafficking were able to predict and or discriminate between individuals with a robust (responder) or minimal (non-responder) inflammatoryresponse to inhaled O3.

We report that in a group of 27 adult volunteers, 18 were robust inflammatory responders with a mean O3-induced increase in % PMNs of 35%, and 9 were non-responders with a mean O3-induced increase in % PMNs of 0.6%. Responders also had significantly elevated levels of pro-inflammatory cytokines IL-8 and IL-1β. At baseline, neither % PMN levels nor gene expression profiles differed between inflammatory responders and non-responders. This was somewhat surprising since we have shown previously that baseline gene expression can be used as an indicator of inter-individual responsiveness to environmental contaminants [22]. One explanation could be the limited selection of genes that were analyzed on the targeted microarray. It is possible that a whole genome approach would uncover baseline gene predictors of inflammatory responsiveness to O3.

A select number of biological indicators did however distinguish the inflammatory phenotypes at baseline. For the responders, these included significantly decreased expression of cell surface phenotypes (CD14, CD86) decreased phagocytosis and elevated IL-8 levels, suggesting that at baseline, individuals with the inflammatory responsive phenotype have muted innate immune function in concert with a primed potential toward PMN influx due to high pre-existing levels of IL-8.

Post exposure, responders had enhanced inflammation as demonstrated by increased PMN, IL-8 and IL-1β levels, and activated immune function in concert with minimal immune cell trafficking-associated genomic signaling. Interestingly, non responders had the opposite post- exposure responses demonstrating unchanged inflammation, down-regulated immune function but with a robust immune cell trafficking genomic response. These data demonstrate the presence of a negative association between a specific innate immune genomic response and immuno-inflammatory functional responses in the airways. Specifically, up-regulation of genes associated with immune cell trafficking appears to be negatively associated with macrophage expression of CD16/FcRγIII and CD11b/CR3, both of which mediate innate immune responses, and neutrophil phagocytosis. It is important to acknowledge that these measurements were conducted in sputum cells that reflect events in the large versus distal airways, a compartmentalized response to ozone. Inflammatory responses are noted to be different in large versus distal airways.

Since our cohort of subjects included asthmatics, we assessed whether disease status conferred an increased likelihood of being an inflammatory responder or non-responder. We found that neither asthma nor atopy (without asthma) significantly predisposed one toward a particular inflammatory phenotype. This suggests that although O3 is associated with increased exacerbation of asthma and can induce greater O3-induced lung function decline in asthmatics versus non-asthmatics [23,24], inflammatory responsiveness to O3 may not be wholly dependent on asthma status or atopy, but when it occurs in asthmatics, may be a risk factor for asthma exacerbation.

Following O3 exposure, there were some common genomic responses between responders and non responders. These included genes that are associated with inflammation, specifically members of the NF-κB pathway. These data support research that has shown that O3 is known to cause increases in NF-κB binding activity in macrophages and type II epithelial cells [25]. Interestingly, while both groups modulated these inflammation-associated genes, it was the O3 non-responders who showed an enrichment for genomic signaling related to immune cell trafficking. Finally, these results identify through a systems biology approach, novel baseline biological indicators and post exposure discriminators of inflammatory responsiveness to O3. Our data highlight the complex interplay between innate immune-associated genomic signaling and immuno-inflammatory functional responses to ozone, factors that likely influence disease susceptibility in inflammatory responsive individuals.

It is important to acknowledge that with these intriguing data, certain limitations were present in this study. Most noteworthy, was the mixed sputum cell population that was used for the gene expression data. Absent a single cell population from which to draw conclusions, the effects of other cell types contributing to the overall genetic signaling cannot be ignored, but in this study for example, neutrophil levels were not likely responsible for the observed differences in genomic signatures.

## Conclusions

Inflammatory responsive and non responsive individuals to O3 each demonstrate an inverse relationship between innate immune genomic signaling and immuno-inflammatory responses to O3, but do so in opposite directions. Inflammatory responders have enhanced immuno-inflammatory function with muted innate immune genomic signaling, while non responders have decreased immuno-inflammatory function with robust innate immune genomic signaling. These distinctions, particularly in genomic signaling, may help elucidate the mechanisms underlying O3-induced morbidity in individuals with differing levels of inflammatory responsiveness to O3.

## Abbreviations

FEV1: Forced expiratory volume in one second; IL: Interleukin; INF: Interferon; MFI: Mean fluorescence intensity; NFκB: Nuclear factor kappa-B; PMN: Polymorphonuclear neutrophil; ppm: Parts per million; TNF: Tumor necrosis factor; O3: Ozone.

## Competing interests

The authors declare that they have no competing interests.

## Authors’ contributions

NEA and DBP conceived of the experiments and were responsible for human subjects recruitment and exposures. DBP was responsible for clinical oversight. NEA was responsible for sample collection and cell isolation and JCL performed cell phenotype analysis. RNA was extracted by JWB. Microarray design and fabrication were performed by JWB and JT. Data were analyzed by RCF, JER, HZ, BZ. The manuscript was written by RCF, JER, DBP and NEA. All authors read and approved the final manuscript.

## Supplementary Material

Additional file 1 Genes significantly differentially expressed after ozone challenge. Click here for file

Additional file 2 Networks constructed using the inflammatory non-responders’ ozone-associated genes. Click here for file

Additional file 3 Networks constructed using the inflammatory responders’ ozone-associated genes. Click here for file
