# Supplementary material for: Individuals with increased inflammatory response to ozone demonstrate muted signaling of immune cell trafficking pathways
Source: Respir Res. 2012 Oct 3;13(1):89. doi: 10.1186/1465-9921-13-89 (PMC3607990; doi:10.1186/1465-9921-13-89)
Supplement: Additional file 1 — Genes significantly differentially expressed after ozone challenge. [file 1465-9921-13-89-S1.pdf]

Additional File 1: Genes significantly differentially expressed after ozone challenge. Genes are sorted according to expression level fold change (FC) (post-ozone challenge / pre-ozone challenge) in each groups.

Symbol	Non-Responders' FC	Responders' FC	Significantly Altered in Non-Responders, Responders, or Both
SERPINE2	3.06	2.10	Both
CDKN3	2.82	1.85	Both
CCND1	2.73	1.87	Both
MMP2	2.64	1.99	Both
SFRP1	2.53	1.91	Both
NCF2	2.42	1.99	Both
STK12	2.22	2.23	Both
PSMB10	736.50	0	Non-Responders
TXN2	249.33	0	Non-Responders
SEMA3B	89.74	0	Non-Responders
FCGR1A	79.33	0	Non-Responders
FBN1	33.65	0	Non-Responders
CCNG1	31.65	0	Non-Responders
API5	23.00	0	Non-Responders
SCUBE1	19.94	0	Non-Responders
CD97	19.59	0	Non-Responders
BRCA1	18.35	0	Non-Responders
HAS1	18.24	0	Non-Responders
LCN2	17.57	0	Non-Responders
BCL3	13.08	0	Non-Responders
GNLY	12.69	0	Non-Responders
PLK2	9.63	0	Non-Responders
NLRP11	9.25	0	Non-Responders
STEAP	8.82	0	Non-Responders
NGB	6.23	0	Non-Responders
TUBA1	5.36	0	Non-Responders
GATA3	5.27	0	Non-Responders
BCL2A1	4.94	0	Non-Responders
CHIT1	4.86	0	Non-Responders
SEPP1	4.78	0	Non-Responders
ALDH1B1	4.46	0	Non-Responders
RELB	4.42	0	Non-Responders
IL1R2	4.29	0	Non-Responders
HLA-C	4.28	0	Non-Responders
NMES1	4.21	0	Non-Responders

Symbol	Non-Responders' FC	Responders' FC	Significantly Altered in Non-Responders, Responders, or Both
LOX	4.01	0	Non-Responders
TBP	4.00	0	Non-Responders
TNFRSF10C	3.99	0	Non-Responders
IL1B	3.98	0	Non-Responders
B2M	3.98	0	Non-Responders
IGFBP2	3.92	0	Non-Responders
S100A12	3.82	0	Non-Responders
SERPINF1	3.77	0	Non-Responders
BATF	3.69	0	Non-Responders
PSMC4	3.63	0	Non-Responders
P2RY5	3.62	0	Non-Responders
CCR9	3.61	0	Non-Responders
SOD2	3.60	0	Non-Responders
PSMC3	3.52	0	Non-Responders
IL8RA	3.50	0	Non-Responders
NCF4	3.46	0	Non-Responders
GTPBP4	3.45	0	Non-Responders
SEMA4C	3.44	0	Non-Responders
SELL	3.43	0	Non-Responders
PTMA	3.42	0	Non-Responders
IFITM2	3.37	0	Non-Responders
S100A8	3.36	0	Non-Responders
HAS3	3.35	0	Non-Responders
LILRB2	3.29	0	Non-Responders
CCL28	3.25	0	Non-Responders
ITGB7	3.21	0	Non-Responders
NGFRAP1	3.20	0	Non-Responders
PRDX4	3.16	0	Non-Responders
PROK2	3.15	0	Non-Responders
ADAM8	3.12	0	Non-Responders
BCL6	3.12	0	Non-Responders
NFKBIA	3.07	0	Non-Responders
TGFBR3	3.05	0	Non-Responders
COL5A1	3.01	0	Non-Responders
ILF2	3.01	0	Non-Responders
ODC1	2.96	0	Non-Responders
GSTM3	2.92	0	Non-Responders
SEMA5A	2.83	0	Non-Responders

Symbol	Non-Responders' FC	Responders' FC	Significantly Altered in Non-Responders, Responders, or Both
ITIH2	2.80	0	Non-Responders
LEAP2	2.77	0	Non-Responders
EXO1	2.76	0	Non-Responders
IGFBP3	2.75	0	Non-Responders
PXMP2	2.69	0	Non-Responders
DHCR24	2.64	0	Non-Responders
CTNNAL1	2.61	0	Non-Responders
AHSG	2.61	0	Non-Responders
BFAR	2.58	0	Non-Responders
TREM1	2.50	0	Non-Responders
GFPT2	2.50	0	Non-Responders
CYC1	2.49	0	Non-Responders
C4A	2.48	0	Non-Responders
HAS2	2.48	0	Non-Responders
IMPDH2	2.44	0	Non-Responders
LILRA3	2.42	0	Non-Responders
COL1A2	2.40	0	Non-Responders
CSF1R	2.38	0	Non-Responders
TFF1	2.38	0	Non-Responders
FCGR3B	2.33	0	Non-Responders
NQO1	2.30	0	Non-Responders
TROAP	2.28	0	Non-Responders
LILRB1	2.20	0	Non-Responders
FST	2.08	0	Non-Responders
PSMB5	2.00	0	Non-Responders
GBP2	1.84	0	Non-Responders
PDCD8	1.81	0	Non-Responders
NCR2	-0.34	0	Non-Responders
ZNF144	-0.47	0	Non-Responders
FAIM2	-0.66	0	Non-Responders
HSPA5	-0.70	0	Non-Responders
IL1F8	-0.99	0	Non-Responders
SLC4A7	-1.28	0	Non-Responders
P2RXL1	-1.33	0	Non-Responders
ENG	-1.79	0	Non-Responders
EGF	-2.12	0	Non-Responders
ISG20	-2.29	0	Non-Responders
GLA	-3.01	0	Non-Responders

Symbol	Non-Responders' FC	Responders' FC	Significantly Altered in Non-Responders, Responders, or Both
NRAS	-3.18	0	Non-Responders
MAPK11	-3.48	0	Non-Responders
SLC44A1	-4.75	0	Non-Responders
RAB2	-5.78	0	Non-Responders
MYC	-6.47	0	Non-Responders
RB1	-6.56	0	Non-Responders
ATRN	-9.17	0	Non-Responders
VAV3	-9.87	0	Non-Responders
AP1S1_HUMAN	-12.18	0	Non-Responders
HYOU1	-13.49	0	Non-Responders
FADD	-14.33	0	Non-Responders
PPP5C	-16.63	0	Non-Responders
UBE2N_HUMAN	-19.34	0	Non-Responders
GTF2F1	-21.66	0	Non-Responders
SLC20A2	-59.69	0	Non-Responders
VCL	-104.18	0	Non-Responders
CD163	0	4.21	Responders
MAL2	0	3.79	Responders
CLDN3	0	3.17	Responders
RBBP7	0	2.79	Responders
CLU	0	2.49	Responders
ASS	0	2.42	Responders
HLA-DRB1	0	2.41	Responders
GAPD	0	2.25	Responders
NMU	0	2.13	Responders
ID1	0	1.97	Responders
MYBL2	0	1.89	Responders
TNC	0	1.85	Responders
TGFB1I1	0	1.79	Responders
