# Supplementary material for: Individuals with increased inflammatory response to ozone demonstrate muted signaling of immune cell trafficking pathways
Source: Respir Res. 2012 Oct 3;13(1):89. doi: 10.1186/1465-9921-13-89 (PMC3607990; doi:10.1186/1465-9921-13-89)
Supplement: Additional file 2 — Networks constructed using the inflammatory non-responders’ ozone-associated genes. [file 1465-9921-13-89-S2.pdf]

Additional File 2: Networks constructed using the inflammatory non-responders' ozone-associated genes.

Network Number	Molecules in Network	p-value
1	ALDH1B1, B2M, BATF, BFAR, Cbp, Cpla2, Fc gamma receptor, FCGR1A, FCGR3B, GFPT2, GLA, Has, HAS1, HAS2, HAS3, HLA Class I, HLA-B27, HLA-C, Ikb, Ikk (family), IL1F8, LILRB1, LILRB2, MHC Class I (complex), MHC CLASS I (family), NFkB (complex), NFkB (family), PLK2, PRDX4, PTMA, RELB, SAA, TGFBR3, TNFRSF10C, UBE2N	1E-43
2	20s proteasome, AIFM1, AURKB, BCL6, Calpain, Caspase, CYC1, Cytochrome c, FADD, GATA3, GNLY, Histone h3, Histone h4, Hsp70, HSPA5, IgG, Igm, IL12 (complex), Immunoglobulin, Interferon alpha, ISG20, Jnk, MYC, NFKBIA, NGFRAP1, NQO1, PPP5C, PSMB5, PSMB10, Rb, RNA polymerase II, SELL, Serine Protease, SFRP1, TXN2	1E-35
3	ADAM8, Alp, C4A/C4B, COL1A2, COL5A1, collagen, Collagen Alpha1, Collagen type I, Collagen(s), CSF1R, Fibrinogen, FST, Growth hormone, Hsp27, HYOU1, Igfbp, IGFBP2, IGFBP3, IL1, JINK1/2, Laminin, Ldh, LDL, LOX, MMP2, PCGF2, Pdgf (complex), PDGF BB, PI3K (complex), S100A12, SERPINF1, Sod, SOD2, STAT5a/b, Tgf beta	1E-26
4	ADCYAP1, ADK, ALDH1B1, APP, ATP, ATRN, C11orf82, cholesterol, DHCR24, dihydrotestosterone, F2, GFPT2, GLA, GULP1, IL1/IL6/TNF, IL1F8, ILF2, IMPDH2, ITGB7, LEAP2, LOC729505, NGB, NLR, P2RX2, P2RX5, P2RX6, PXMP2, Rac, RELA, RPL27, SEPP1, SLC16A5, SLC4A7, TNF, TRAFD1	1E-23
5	ABCC6, API5, CCR9, CCRL2, CD97, CSF1, CXCR1, DGKA, EXO1, FAIM2, FGF2, GPR37, GPR44, GPR146, HNF4A, HTR1E, hyaluronic acid, IFITM2, IFNA2, IL2, ITIH2, ITIH3, JKAMP, KRT8, LPAR6, NCR2, PSMC3, ROBO3, SEMA3B, SLC19A2, SLC44A1, TP53, TRO, TROAP, VEGFA	1E-23
6	AHSG, Akt, Ap1, BCL2A1, CCND1, CDKN3, Creb, Cyclin E, DHCR24, FAIM2, GBP2, Gm-csf, GTPBP4, hCG, Ifn gamma, MAP2K1/2, MAPK11, Mek, NCF2, NCF4, Nfat (family), NfkB1-RelA, NRAS, P38 MAPK, Pld, PROK2, Raf, Ras, Ras homolog, Sapk, STEAP1, TCR, Tnf, TSH, Vegf	1E-21
7	26s Proteasome, Actin, Calmodulin, Ck2, EGF, ENG, ERK, Estrogen Receptor, F Actin, FSH, G protein alphai, Gpcr, Gsk3, GST, GSTM3, Ifn, Insulin, Lh, Mapk, Mmp, ODC1, Pka, Pkc(s), PLC, PLC gamma, PP2A, PSMC4, RAB2A, RB1, Rock, S100A8, SERPINE2, SLC20A2, TUBA4A, VCL	1E-19
8	AP1S1, BCL3, BRCA1, Cbp/p300, CCNG1, Cdc2, Cdk, Ctbp, Cyclin A, Cyclin D, E2f, ERK1/2, GC-GCR dimer, Gcn5l, Gelatinase, GTF2F1, Hat, Hdac, HISTONE, Holo RNA polymerase II, IL1R2, LCN2, N-cor, RNA polymerase I, Rxr, Smad2/3-Smad4, Sos, SWI-SNF, TBP, TFF1, TFIIA, TFIIF, Thyroid hormone receptor, TREM1, VAV3	1E-17
9	Ccl9, CCL24, CCL25, CCL27, CCL28, CCR9, CCRL2, CDH22, CEBPB, CHEMOKINE, CHIT1, CTNNAL1, CTNNB1, CXCL16, CYSLTR1, DEFA5, FBN1, FZD6, GHSR, GIPC2, heparin, IKK (complex), IL12 (family), IL1B, MYOC, PLB1, RHOA, SCUBE1, SCUBE2, SEMA4C, SEMA5A, SEPP1, SLC37A4, TMEM176B, Ubiquitin	1E-14
