# Supplementary material for: Individuals with increased inflammatory response to ozone demonstrate muted signaling of immune cell trafficking pathways
Source: Respir Res. 2012 Oct 3;13(1):89. doi: 10.1186/1465-9921-13-89 (PMC3607990; doi:10.1186/1465-9921-13-89)
Supplement: Additional file 3 — Networks constructed using the inflammatory responders’ ozone-associated genes. [file 1465-9921-13-89-S3.pdf]

Additional File 3: Networks constructed using the inflammatory responders' ozone-associated genes.

Network Number	Molecules in Network	p-value
1	Akt, Androgen-AR, ASS1, AURKB, CCND1, CDKN3, CLDN3, CLU, Collagen type I, ERK, ERK1/2, Estrogen Receptor, ETS-ELK1, GAPDH, Histone h3, Histone h4, HLA-DRB1, ID1, Immunoglobulin, Insulin, Jnk, Mapk, MMP2, MYBL2, NCF2, NFkB (complex), P38 MAPK, PDGF BB, RBBP7, RNA polymerase II, SFRP1, Tgf beta, TGFB1I1, TNC, Vegf	1E-44
2	7S NGF, APCS, BTG2, CD163, CDKN2C, CP, CXCL5, FSTL1, Gm-csf, GPI, HERC5, HP, IGFBP6, IL6, JINK1/2, KLKB1, nicotinic acid, NMU, PLAA, PROC, RRM2, S100A7, S100A12, SAA1, SERPINA3, SERPINE2, SMPD2, stearic acid, THBD, Timp, TNF, TNFRSF12A, TNFRSF6B, TNFSF12, VLDLR	1E-06
